# Duplication of Left Anterior Descending Artery: A Case Report on a Rare Abnormality

**DOI:** 10.7759/cureus.59764

**Published:** 2024-05-06

**Authors:** Tushar Kalekar, Apurvaa Pachva, Sai Pavan Kumar

**Affiliations:** 1 Department of Radiology, Dr. D. Y. Patil Medical College, Hospital and Research Centre, Dr. D. Y. Patil Vidyapeeth, Pune, IND

**Keywords:** left anterior descending coronary artery (lad), coronary anomalies (cas), ischemic heart disease, coronary artery disease (cad), computed tomography coronary angiography

## Abstract

Duplication of the left anterior descending coronary artery (LAD) is a benign condition. The formation of a double LAD is a rare phenomenon among coronary artery anomalies. The categorization of the branching pattern of the LAD has been articulated well in numerous studies, owing to the widespread adoption of computed tomography angiography. Anomalous coronaries are a crucial pathological condition that should be examined. Individuals who are suffering from chest pain should be aware that it can potentially lead to myocardial ischemia, arrhythmia, or sudden cardiac death. Here is a unique case study detailing the diagnosis of dual LAD in a 50-year-old female patient.

## Introduction

Coronary artery anomalies are rare contributors to chest pain and have the potential to result in sudden death [1]. The LAD follows a consistent path of the main coronary artery. The anterior interventricular sulcus contains the LAD artery, a branch of the left major coronary artery that runs diagonally toward the heart's apex. Duplication of the left anterior descending artery (LAD) is a rare abnormality.

Spindola-Franco et al. [2] first described double LAD and classified it into four subtypes in 1983. The presence of two LAD branches is referred to as double LAD. The initial division, referred to as the short LAD, ends at a high point within the anterior interventricular groove. Types 1-3 of the main LAD are typically the source of the second branch, known as the long LAD; however, type 4 of the right coronary artery (RCA) can occasionally give rise to anomalous development of the second branch. As aberrant coronaries have the potential to cause myocardial ischemia, arrhythmia, or sudden cardiac death, they are a crucial clinical entity that should be looked at in patients presenting with chest discomfort [2,3]. This report presents a rare case of a 50-year-old female patient with a duplication of the LAD artery.

## Case presentation

A middle-aged female patient visited the cardiac outpatient clinic with a chief complaint of retrosternal chest pain persisting for the past three months, accompanied by palpitations. The patient additionally reported experiencing dyspnea during physical exercise. The patient has had a documented history of both diabetes and hypertension for the past decade. The electrocardiogram (ECG) revealed normal sinus rhythm along with the presence of T-wave inversions in anterior leads V2 and V6. The 2D echocardiography results indicated a normal ejection fraction of 60%, hypokinesia of the anterior septum and apex, normal chamber dimensions, normal cardiac valves, and minimal pericardial effusion without any signs of cardiac tamponade. The troponin I and creatine phosphokinase-MB results were positive. Based on the aforementioned findings, the patient was diagnosed with acute coronary syndrome. The patient was referred to the radiology department for computed tomography coronary angiography (CTCA). Informed content was taken.

We performed a CTCA on an Ingenuity 128 CT scanner by Philips (Fort Myers, FL). The approach employed was a helical scan with a slice measuring 0.8 x 0.8 mm, a field of view of 200-300 nm, and a pitch value of -0.2. The rotation lasted 0.41 seconds, and the kilovoltage was set to 120. The auto-milliampere-seconds feature was utilized. Intravenous Iomeron 400 of 1.5 mL/kg with 80-100 mL contrast was injected into the patient via bolus tracking. ECG gating is performed based on heart rate. The retrospective reconstruction method was performed using slices ranging from 0.4 to 10 mm in thickness. The calcium score measured approximately 160, with the LAD measuring 157.6 and the circumflex artery measuring 2.5. Left dominance is noted. The left main artery appears to be normal in both its course and diameter, and there is no severe narrowing. The left circumflex artery exhibits dominance.

**Figure 1:**
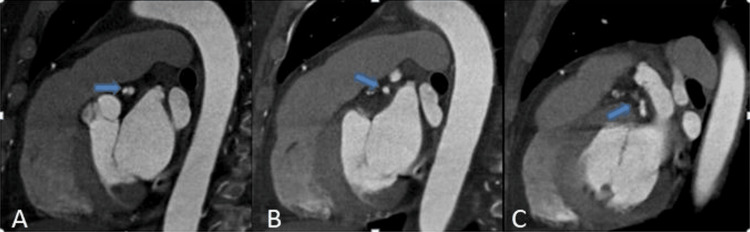
CTCA showing a large calcified mixed density plaque/thrombus in proximal (A), mid (B), and distal (C) parts of LAD causing >90% thrombosis (blue arrows) CTCA: computed tomography coronary angiography; LAD: left anterior descending artery

A large calcified mixed-density plaque or thrombus with a CT attenuation of 40-50 Hounsfield units and measuring 2.5 cm in length is causing more than 90% thrombosis in the proximal LAD (Figures 1A-1C).

**Figure 2:**
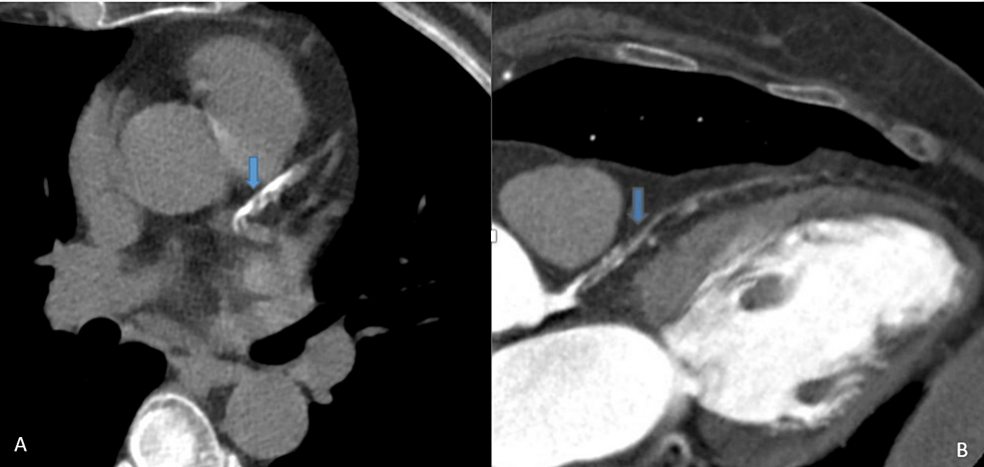
(A) Noncontrast CTCA and (B) contrast CTCA, showing a large calcified mixed density plaque/thrombus from left main bifurcation to the origin of LAD and to the origin of D1. Beyond that, hypoperfusion is noted in LAD (blue arrows) CTCA: computed tomography coronary angiography; LAD: left anterior descending artery

This thrombosis extends from the left main bifurcation to the origin of the LAD and extends further to the origin of D1. Beyond this point, there is evidence of reduced blood flow (hypoperfusion) in the LAD (Figures 2A, 2B).

The RCA is of typical size and has a normal path. The marginal and other diagonal arteries, as well as the posterior descending artery and posterior left ventricular artery, are within typical parameters.

**Figure 3:**
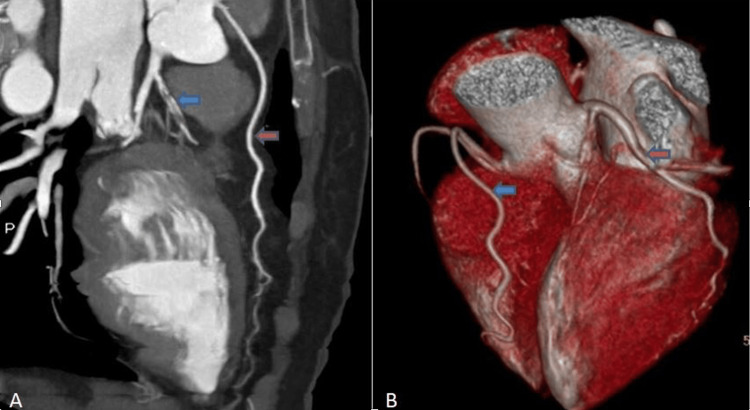
(A) Reformatted CTCA and (B) volume rendering technique images, showing thrombosed LAD (blue arrows) and accessory LAD (red arrows) CTCA: computed tomography coronary angiography; LAD: left anterior descending artery

An additional artery, measuring 2.9 mm in diameter, has been identified as originating from the right coronary cusp adjacent to the RCA. The artery is visible, passing through the right ventricular outflow tract and the aortic root. It then crossed to the left side, above the main pulmonary artery, and continued to supply the anterior interventricular groove. This is an accessory or duplicated LAD (Figures 3A, 3B).

This artery was observed extending to the heart apex, mostly the accessory LAD. The results were confirmed using catheter angiography, which revealed a left-dominant system with a type III vessel and an ostioproximal location. Mild plaque, 95% stenosis, and 99% subtotal blockage in the proximal segment were observed, and a good-caliber accessory LAD supplied the anterior septum. Percutaneous coronary angioplasty was used to implant two stents in the patient's main LAD artery as part of their treatment.

## Discussion

Dual LAD refers to a set of abnormalities in which the LAD either divides into two primary branches or originates from two separate origins to serve the same area in separate sections [3]. The prevalence of double LAD coronary artery is seen to range from 0.68% to 6% in various case groups [4]. This condition is consistently misdiagnosed during coronary angiography. In order to avoid any ambiguity in coronary angiography and reduce difficulties in coronary procedures, it is crucial to know different types of double LAD [2].

Spindola-Franco et al. [2] first diagnosed LAD artery abnormalities in 1983, employing the coronary catheter angiography technique. Three varieties of LADs exist, each with a small branch that ends at a high position within the anterior interventricular groove. In addition, there is a lengthy branch that usually begins as an early branch. In type 4 cases, the RCA originates at the LAD [2].

Gaining a comprehensive understanding and recognizing the existence of LAD is of utmost importance for several reasons. Precisely comprehending the anatomical characteristics of coronary arteries is essential for devising effective strategies for surgical vascularization. For instance, if just the abbreviated LAD is transplanted, it is crucial for the surgeon to know the anatomical characteristics in order to accurately expose the vessel at a higher position than typical in the anterior interventricular groove. A thorough understanding of different types of double LAD is crucial in order to prevent the wrong positioning of an arteriotomy and to ensure the proper revascularization of the appropriate blood vessel [5].

If there is a substantial narrowing in both the short and long LADs, it may be necessary to perform grafts on both channels. This is due to the fact that these two distinct blood vessels are responsible for supplying blood to the septum and wall of the left ventricle [2]. Furthermore, because of the difficulty in visualizing the additional vessel, especially if the LAD arises from the right coronary sinus, there is a potential for misinterpreting the unusual anatomical characteristics during conventional coronary angiography as a blockage in the middle section of the LAD. The lack of attention to this detail can result in unusual and apparently conflicting discoveries of blockages in the coronary arteries and irregularities in the movement of specific sections of the heart wall. This is because, in the majority of cases, the main septal perforators receive their blood supply primarily from the short LAD. At the same time, the LAD proper is responsible for supplying blood to the major diagonal veins. Spindola-Franco et al. [2] conducted a study where they observed the occlusion of the short LAD in a type 1 dual LAD configuration, whereas the regular LAD and the long LAD were not affected. This resulted in the anterior interventricular septum being immobile while the anterior left ventricular wall continued to move normally.

In their prospective investigation, Nikolić et al. [6] examined approximately 3,000-3,500 hearts, incorporating autopsies. They identified 10 occurrences of a type 3 double LAD. The researchers found that these atherosclerotic alterations were more pronounced and severe in the shorter double LAD than in the longer one. Moreover, it has been established that myocardial bridging exerts a safeguarding impact on the type 3 dual LAD and has a role in the progression of atherosclerosis in both the short and long segments of the LAD, taking into account the influence of both modifiable and nonmodifiable variables.

## Conclusions

CT interpreters must be able to recognize the existence of double LAD during cardiac CT images. Detecting an additional LAD is critical for correct diagnosis and treatment planning. An aberrant coronary artery's course can be difficult to establish with angiography alone, especially if it passes between the major vessels or through the myocardium. CT coronary angiography can provide spatial information on the long LAD and its surrounding structures. Recognizing this anomaly is critical for complete revascularization in the presence of existing coronary artery disease and preventing abrupt cardiac death.
